# A case report: deep and durable response to low-dose lenvatinib and tislelizumab in an elderly patient with advanced intrahepatic cholangiocarcinoma

**DOI:** 10.3389/fphar.2024.1447582

**Published:** 2024-09-26

**Authors:** Pei Zhang, Xin Wang, Ruizhen Li, Xiaoying Li, Ke Cheng, Dan Cao

**Affiliations:** Division of Abdominal Tumor Multimodality Treatment, Cancer Center, West China Hospital, Sichuan University, Chengdu, Sichuan, China

**Keywords:** intrahepatic cholangiocarcinoma, lenvatinib, tislelizumab, elderly patient, deep and durable response

## Abstract

**Background:**

Older patients with advanced cholangiocarcinoma lack systemic therapy standards. These people have a high risk of chemotherapy, accompanied by adverse reactions and even discontinuation of treatment.

**Case presentation:**

We report a 78-year-old female subject with advanced intrahepatic cholangiocarcinoma presenting with unresectable lesions involving the hepatic veins, along with extensive metastatic lymph nodes. After the geriatric assessment, capecitabine was utilized for only one cycle owing to adverse events (AEs). Next, a combination of low-dose lenvatinib and tislelizumab was administrated as a second-line treatment, which resulted in remarkable early tumor shrinkage. The following individual lenvatinib taper enabled a manageable safety profile and durable deep response. A near-complete response was achieved, with the primary tumor significantly reducing from 5.6 cm × 4.7 cm to nearly complete disappearance, accompanied by complete regression of lymph nodes, and both progression-free survival and overall survival exceeding 24 months.

**Conclusion:**

The case provides valuable insights that could influence future treatment strategies for older patients with advanced cholangiocarcinoma who are unsuitable for chemotherapy. The dose-individualized chemotherapy-free regime of lenvatinib and tislelizumab might be used in similar cases to improve their outcomes.

## Background

Cholangiocarcinoma has a poor prognosis, with a 5-year OS rate of less than 20% (Raggi et al., 2022). The combination of gemcitabine and cisplatin is currently the first-line standard treatment for advanced biliary system tumors (Valle et al., 2010). Adding PD-1/PD-L1 immunotherapy to chemotherapy further benefits patient survival (Oh et al., 2022; Kelley et al., 2023). However, data from patients older than 75 years are relatively scarce. Another study notes that older patients have more chemotherapy side effects, making them more likely to undergo dosage modification or discontinue treatment (Kalsi et al., 2014). Therefore, introducing geriatric assessment and exploring chemotherapy-free options becomes particularly important (Hurria et al., 2011). The combination of PD-1 inhibitors and VEGFR-TKIs represents a promising therapeutic strategy. Tislelizumab, in combination with lenvatinib, has demonstrated notable antitumor activity with a favorable safety profile in hepatocellular carcinoma (Xu et al., 2024). Additionally, emerging small-scale studies suggest potential efficacy in cholangiocarcinoma (Zhang et al., 2021). Against this background, we report an elderly patient diagnosed with advanced cholangiocarcinoma who received individualized dose-reduced combined targeted therapy and immunotherapy, exhibiting both safety and durable deep tumor remission, thereby offering a chemotherapy-free option for elderly patients with cholangiocarcinoma.

## Case presentation

**FIGURE 1:**
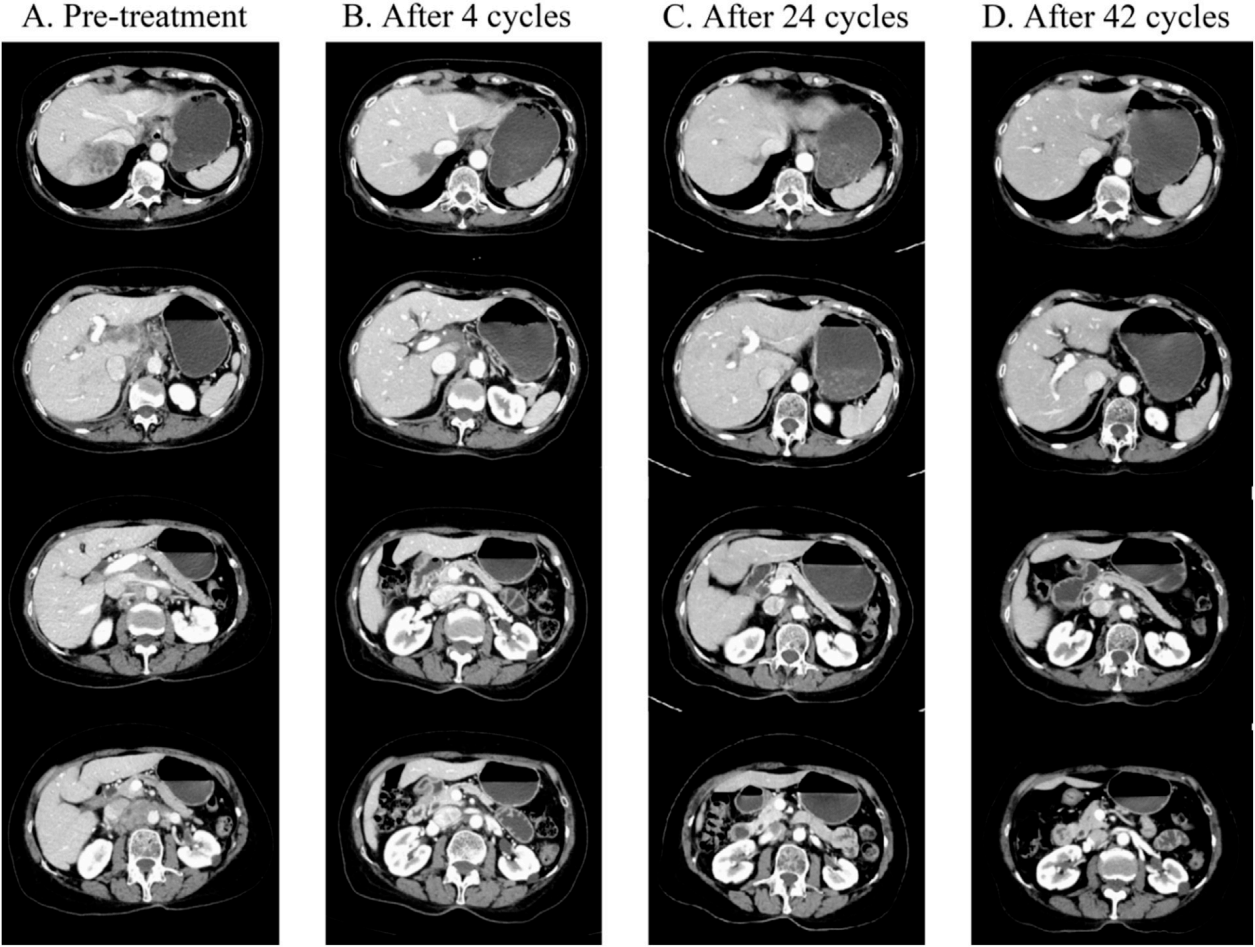
**(A)** Prior to treatment, CT showed a soft tissue mass located in the right lobe of the liver (size 5.6 cm × 4.7 cm), with increased and enlarged lymph nodes in the perigastric, hepatoportal area, hepatogastric ligament, and para-abdominal aorta, which were partially fused, and most of them were metastatic from lymph nodes (2021-07-12). **(B)** CT showed that after four cycles of treatment, compared with the 2021-07-12 CT, the primary tumor in the right lobe of the liver was significantly reduced (size 3.1 cm × 3.0 cm), and the caudate lobe lesion was a new metastasis. The hepatic portal area and hepatogastric ligament, cardiophrenic angle area, and para-abdominal aorta metastasis were significantly reduced compared to the previous area. **(C)** CT on 2022-11-29 showed that after 24 courses of treatment, the primary tumor in the right lobe of the liver almost disappeared, the residual low-density lesions were most likely to be necrotic, and some metastases in the liver almost disappeared. Compared with before, the size of the hepatic portal area and hepatic and gastric ligaments, cardiopulmonary angle area, and para-abdominal aortic lymph nodes continued to shrink. **(D)** CT on 2024-01-05 shows that after 42 courses of treatment, all lesions have almost completely disappeared compared with 2022-11-29 CT.

A 78-year-old woman presented with upper abdominal pain. Subsequent contrast-enhanced computed tomography (CT) scan in July 2021 revealed a 5.6 cm × 4.7 cm mass in the right hepatic lobe, with extension into the right hepatic vein. Additionally, swollen lymph nodes were observed around the stomach, portal area, hepatogastric ligament, and para-aortic region, encircling the superior mesenteric arteries and veins (Figure 1). Poorly differentiated adenocarcinoma was confirmed by biopsy, and immunohistochemistry displayed CK7 (+), PCK (+), GPC3 (weak, +), and Ki-67 (+, ∼80%), with other markers being negative, including HER-2 (0). Elevated CA19-9 (42.10 U/mL) and alpha-fetoprotein (AFP) (11.30 ng/mL) were tested, with PIVKA-II under the normal range. The patient had a history of atrial tachycardia and heart failure and underwent radiofrequency ablation without a hepatitis history. Next-generation sequencing (NGS) analysis of the tumor identified MET amplification, low tumor mutational burden (TMB-L), and microsatellite stability (MSS) (Tables 1, 2). Consequently, the patient was diagnosed by the multidisciplinary team (MDT) with unresectable intrahepatic cholangiocarcinoma with multiple abdominal lymph node metastases, classified as clinical stage cT2N1M0 IIIB.

**TABLE 1:** Copy number variation detection results.

Gene	Transcription book	Variation type	Functional area	Number of copies
MET	NM_000245.2	Amplification	All exons	7
JUN	NM_002228.3	Amplification	All exons	4.4

**TABLE 2:** Genome variation detection results.

Gene	Transcription book	Base change	Amino acid change	Functional area	Mutation frequency (%)
TP53	NM_000546.5	c.812A>T	p.E271V	EX8	13.5
EP300	NM_001429.3	c.785G>T	p.G262V	EX3	9.1
HGF	NM_000601.4	c.337G>C	p.G113R	EX3	8.6
SMARCA4	NM_003072.3	c.3555A>T	p.Q1185H	EX26	8.1
CDH23	NM_022124.5	c.7847A>G	p.N2616S	EX55	4.7
TP53	NM_000546.5	c.392A>T	p.N131I	EX5	4.1
ERBB2	NM_004448.2	c.874G>T	p.G292C	EX7	3.9
ABL2	NM_007314.3	c.2222C>T	p.A741V	EX12	3.5

The geriatric assessment score of 5 categorizes the patient as low risk for chemotherapy (Table 3) (Hurria et al., 2011), with favorable performance status (Karnofsky Performance Status (KPS) of 80, Eastern Cooperative Oncology Group (ECOG) PS of 1). Without chemotherapy contraindications, capecitabine (1,500 mg orally twice daily from day 1 to 14) and tislelizumab (200 mg intravenous on day 1, every 3 weeks) were administered as the first-line treatment, with each treatment cycle lasting 3 weeks. However, after only one cycle, the patient discontinued chemotherapy due to grade 2 hand-foot syndrome, fatigue, and diarrhea. The grade 2 hand–foot syndrome, fatigue, and diarrhea observed in this patient are consistent with those reported in other studies involving capecitabine and tislelizumab in similar age groups (van Beek et al., 2018; Zhu et al., 2022).

**TABLE 3:** The results of the geriatric assessment score.

Risk factor	Score
Age >72 years	2
Cancer type GI or GU	0
Chemotherapy dosing, standard dose	2
No. of chemotherapy drugs, polychemotherapy	0
Hemoglobin <11 g/dL (male), 10 < g/dL (female)	0
Creatinine clearance (Jelliffe, ideal weight) < 34 mL/min	0
Hearing, fair or worse	0
No. of falls in last 6 months, one or more	0
IADL: Taking medications, with some help/unable	0
MOS: Walking one block, somewhat limited/limited a lot	0
MOS: Decreased social activity because of physical/emotional health, limited at least sometimes	1

Subsequently, a reduced-dose lenvatinib (4 mg orally daily) combined with tislelizumab (200 mg intravenous every 3 weeks) was administered as a second-line treatment in August 2021.

After four cycles (October 2021), the hepatic lesion shrank with substantially reduced enhancement, and significant regression of abdominal lymph nodes was observed, achieving a partial response (PR), as illustrated in Figure 1. Concurrently, the patient experienced grade 2 diarrhea. A study indicated that if persistent or intolerable grade 2 or 3 AEs occur during lenvatinib treatment, therapy should be paused until symptoms improve, then resumed at the same or a lower dose (Kim et al., 2022). Therefore, we modified the dose to 4 mg every other day, maintaining efficacy while reducing adverse effects.

Under the following repeated cycles, the lesions continuously decreased in size (Supplementary Figure S1), ensuring good efficacy and satisfactory quality of life (QOL). Consequently, the patient declined further local invasive treatments.

**FIGURE 2:**
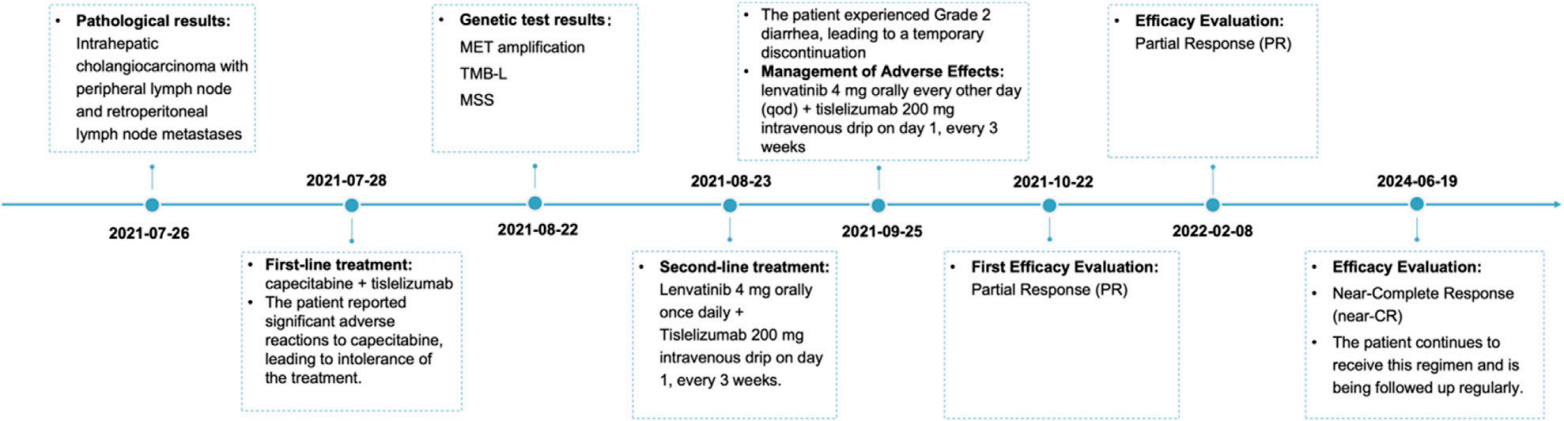
Schematic representation of the course of antitumor therapy. First-line treatment consisted of capecitabine (1,500 mg po bid d1-14) plus tislelizumab (200 mg ivgtt d1, q3w), which was not tolerated by the patient. Subsequently, it was changed to lenvatinib (4 mg po qd) plus tislelizumab (200 mg ivgtt d1, q3w). After one cycle, the patient developed second-degree diarrhea, and the dose was reduced to lenvatinib (4 mg po qod) plus tislelizumab (200 mg ivgtt d1, q3w), and the efficacy was PR after three cycles of the reduced treatment; the efficacy is still PR to date. QOL: quality of life; po: per os (oral); bid: bis in die (twice a day); d1-14: day 1–14; ivgtt: intravenous drip; d1: day 1; q3w: every 3 weeks; qd: quaque die (every day); qod: every other day; PR: partial response.

By the 24th treatment cycle (November 2022), over 90% decrease in liver lesion size and complete disappearance of abdominal lymph nodes were observed. Lenvatinib (4 mg every other day) plus tislelizumab (200 mg every 3 weeks) were administered as maintenance therapy. By January 2024, the patient had achieved near-complete response (near-CR), as illustrated in Figure 1, with a duration of response (DOR) close to 26 months and a progression-free survival (PFS) exceeding 28 months. A series of serum tumor markers, including CEA, CA199, and AFP, were monitored throughout the treatment period. All markers remained relatively stable, with no significant fluctuations (Supplementary Figure S2). The patient was still receiving the regimen as of follow-up in June 2024 (Figure 2). Throughout the second-line therapy, the patient experienced grade 2 thrombocytopenia and grade 1 rash, both effectively managed with proper intervention.

## Discussion

In recent years, with the introduction of immune checkpoint inhibitors, the treatment prospects for cholangiocarcinoma have significantly improved. The results of the TOPAZ-1 and Keynote 966 studies showed that combining PD-1/PD-L1 inhibitors with gemcitabine and cisplatin significantly improves OS in advanced biliary tract cancer (Oh et al., 2022; Kelley et al., 2023). However, clinical data on elderly cholangiocarcinoma patients remain limited. Elderly patients face unique challenges in terms of immune function and tolerance, leaving us with little knowledge about the effectiveness and safety of treatments in this population (Pawelec, 2019; Zhang et al., 2023). Therefore, research on treatments for elderly cholangiocarcinoma patients urgently needs to be strengthened, and even case reports on elderly patients are of great interest.

Elderly patients with cholangiocarcinoma typically have lower tolerance and high requirements for QOL, making traditional chemotherapy regimens potentially unsuitable. Chemotherapy is not contraindicated for elderly patients, but previous literature emphasizes the importance of geriatric assessment (Hurria et al., 2011). Therefore, we conducted a thorough geriatric assessment for this elderly female patient before chemotherapy, and her score was 5. After excluding chemotherapy contraindications, we chose the single-agent chemotherapy capecitabine combined with immunotherapy as the first-line treatment. However, the patient could not tolerate the toxicity of capecitabine and refused further chemotherapy, so we switched to targeted therapy combined with immunotherapy. In targeted therapy, isocitrate dehydrogenase (IDH) 1 inhibitors and fibroblast growth factor receptor (FGFR) inhibitors have been approved by the Food and Drug Administration for cholangiocarcinoma patients with certain genetic mutations (Lodl et al., 2023). However, most patients cannot choose precision-targeted therapy based on genetic mutations. Many clinical studies of anti-angiogenic targeted drugs combined with PD-1/PD-L1 antibodies are underway (Hack et al., 2020). For patients without driver gene mutations or without microsatellite instability-high (MSI-H) status, anti-angiogenic TKIs enhance synergy with immunotherapy through mechanisms such as inhibiting angiogenesis and improving T-cell infiltration in the tumor microenvironment (Lee et al., 2020). These treatment options should be tailored based on the patient’s specific genetic characteristics and condition.

There are few reports on the application of lenvatinib combined with tislelizumab in cholangiocarcinoma (Ding et al., 2021). However, this combination therapy has shown significant antitumor activity and manageable toxicity in patients with unresectable hepatocellular carcinoma (Xu et al., 2023). A study evaluating lenvatinib combined with toripalimab as a first-line treatment for advanced intrahepatic cholangiocarcinoma demonstrated an objective response rate (ORR) of 32.3%, with good tolerability (Jian et al., 2021). Another study investigating lenvatinib combined with sintilimab as a second-line treatment in chemotherapy-refractory advanced intrahepatic cholangiocarcinoma patients presented an ORR of 93.8% in those with high PD-L1 expression (TPS≥10%) (Ding et al., 2022). Additionally, a study enrolled 40 patients of lenvatinib combined with a PD-1 inhibitor as a second-line therapy and reported a disease control rate of 75.0%, with manageable treatment-related AEs (Xie et al., 2022). Based on these findings, our study further emphasizes the potential value of lenvatinib combined with tislelizumab in elderly patients, particularly those over 75 years old, highlighting the importance of individual dose adjustments in the clinical management of this age group.

Noteworthily, although lenvatinib was administrated at an extreme dose (4 mg every other day), a deep and durable response was observed, with achieved PFS for nearly 3 years, whether the combination treatment as maintenance therapy could be used as a stop-and-go strategy with low dosage lenvatinib or drug withdrawal. Based on evidence from metastatic colorectal cancer, a maintenance strategy provides significant clinical benefits compared to complete drug holidays or continued treatment (Esin and Yalcin, 2016). This patient received combination treatment as maintenance therapy at a low dosage for nearly 3 years without intolerable toxicity. Similarly, researchers have reported that another patient with advanced intrahepatic cholangiocarcinoma showed a dramatic response to first-line therapy and a PD-1 inhibitor combined with capecitabine as maintenance therapy, resulting in ongoing PFS (Wang et al., 2021). Therefore, the optimal strategy for maintenance therapy should still be individualized, even when using chemotherapy-free regimens.

Changes in metabolism and the immune system may alter the pharmacokinetics of lenvatinib in elderly patients, potentially enhancing its efficacy (Wildiers et al., 2003). Additionally, tumors that appear hypervascular on imaging indicate a high dependency on angiogenesis, making them more likely to be sensitive to lenvatinib, which could result in a better therapeutic response (Yamamoto et al., 2014; Huang et al., 2021). Moreover, lenvatinib can modify the tumor microenvironment, facilitating T-cell infiltration and synergizing with PD-1 inhibitors (Lu et al., 2021; Kato et al., 2019). These mechanisms may contribute to the sustained antitumor effects observed even at low doses.

Additionally, locoregional therapies such as liver resection, radiofrequency ablation, microwave ablation, radiotherapy, transarterial chemoembolization, etc., were used in unresectable liver-only or liver-dominant intrahepatic cholangiocarcinoma (Owen et al., 2023). MDT suggested stereotactic body radiotherapy could be used as a suitable locoregional treatment after deep PR was achieved, whereas invasive treatments were refused by the patient.

Among elderly patients with advanced disease, especially highly malignant tumors like cholangiocarcinoma, maintaining independence and QOL were more highly valued than survival or temporary tumor regression. Management of adverse events in patients treated with immunotherapy or targeted therapy was crucial. Fortunately, this elder presented mild treatment-related adverse events, grade 2 diarrhea, and fatigue, and these symptoms were significantly relieved after dose reduction. Individual dosage modification reduces the risk during medication in elderly patients.

## Conclusion

Elderly patients lack standardized treatment protocols, making personalized therapy particularly important. The combination of lenvatinib and tislelizumab showed a deep and durable response, as well as prolonged survival in this patient. After experiencing side effects, individualized dose reduction is crucial, and low-dose treatment ensures efficacy without detrimental impact on QOL.

Lenvatinib and tislelizumab were effective and safe. For further validation, cohort studies or randomized controlled trials with large samples are needed. Trials should include patients with other tumors, older patients, and patients intolerant to chemotherapy. Furthermore, long-term follow-up in similar patients is needed to confirm therapy efficacy and monitor late-onset AEs and resistance.

## Data Availability

The original contributions presented in the study are included in the article/Supplementary Material; further inquiries can be directed to the corresponding authors.

## Abbreviations

AE, adverse event; AFP, alpha-fetoprotein; CA19-9, carbohydrate antigen 19-9; CR, complete response; DOR, duration of response; ECOG PS, eastern cooperative oncology group performance status; FGFR, fibroblast growth factor receptor; IDH, isocitrate dehydrogenase; KPS, Karnofsky performance status; MDT, multidisciplinary team; MET, mesenchymal–epithelial transition factor; MSI-H, microsatellite instability-high; MSS, microsatellite stability; NGS, next-generation sequencing; ORR, objective response rate; OS, overall survival; PD-1, programmed death-1; PD-L1, programmed death-ligand 1; PFS, progression-free survival; PIVKA-II, protein induced by vitamin K absence or antagonist-II; PR, partial response; QOL, quality of life; SBRT, stereotactic body radiotherapy; TKI, tyrosine kinase inhibitor; TMB-L, tumor mutational burden-low; TPS, tumor proportion score.
