# Supplementary material for: A case report: deep and durable response to low-dose lenvatinib and tislelizumab in an elderly patient with advanced intrahepatic cholangiocarcinoma
Source: Front Pharmacol. 2024 Sep 26;15:1447582. doi: 10.3389/fphar.2024.1447582 (PMC11464426; doi:10.3389/fphar.2024.1447582)

Figure 2

The figure shows the trends of three tumor markers (CA-199, AFP, and CEA) over time in a patient undergoing treatment. (A) This graph shows the change in CA-199 (u/ml) levels between July 2021 and June 2024. (B) This graph shows the change in AFP (ng/ml) levels between July 2021 and June 2024. (C) This graph shows the change in CEA (ng/ml) levels between July 2021 and June 2024.

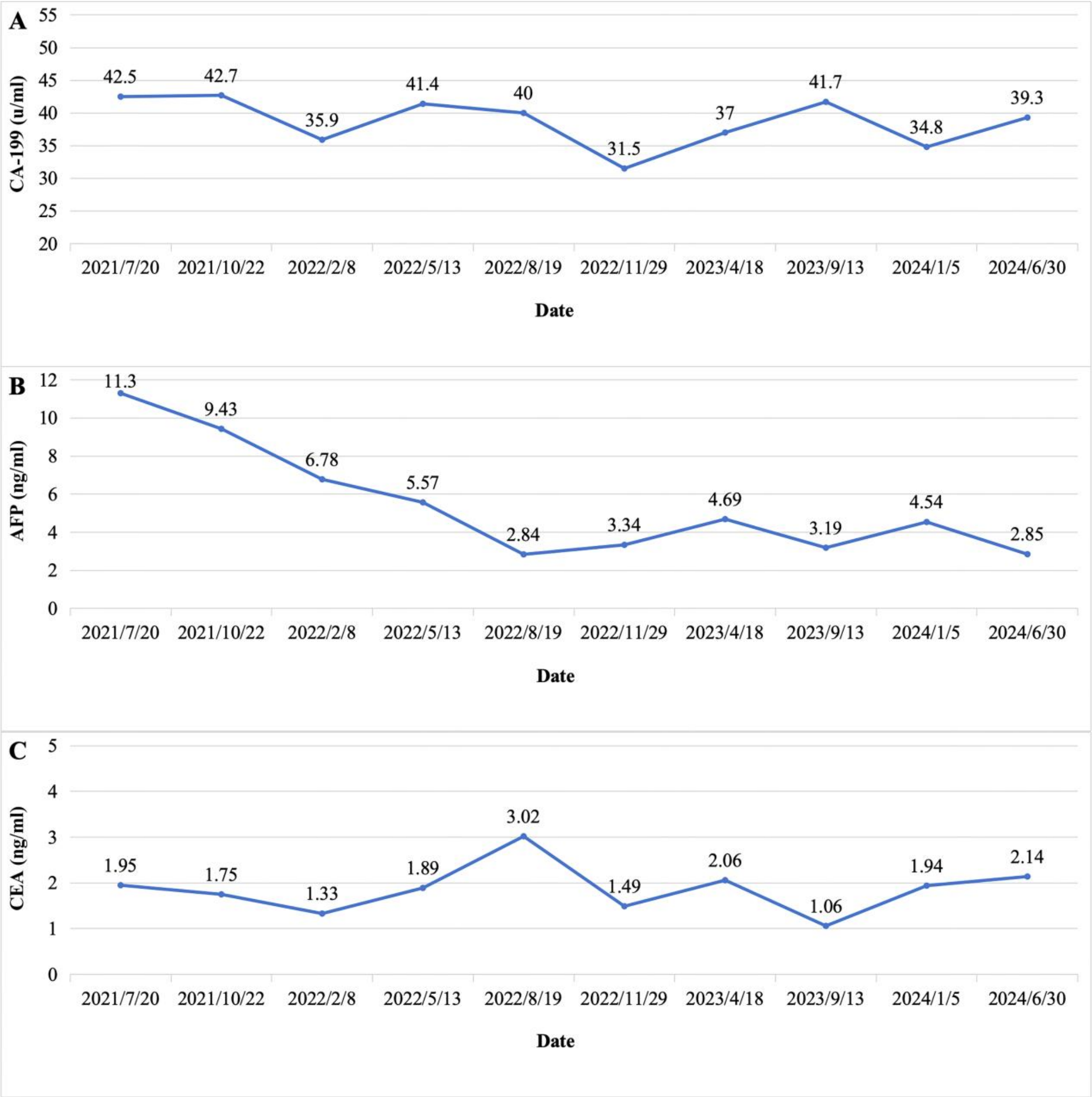

Supplement: Supplementary file 1 [file Image2.pdf]
