# Supplementary material for: A case report: deep and durable response to low-dose lenvatinib and tislelizumab in an elderly patient with advanced intrahepatic cholangiocarcinoma
Source: Front Pharmacol. 2024 Sep 26;15:1447582. doi: 10.3389/fphar.2024.1447582 (PMC11464426; doi:10.3389/fphar.2024.1447582)

Supplementary Material

Figure 1

(A) After 10 cycles of treatment, a CT scan from 2022-02-08 showed that the main tumor in the right lobe of the liver (2.6x2.0 cm) and the caudate lobe lesion had shrunk in the hilar area and hepatogastric ligament, as well as the cardiophrenic angle area and the parabasal abdominal aorta lymph nodes (compare with 2021-10-22). (B) 2022-05-13 CT showed that after 13 cycles of treatment, the primary tumor in the right lobe of the liver had shrunk (size 2.6x1.3 cm), and some of the intrahepatic metastases had shrunk in the hepatoportal area, hepatogastric ligament, cardiophrenic angle area, and the parabasal abdominal aorta lymph nodes continued to decrease in size compared to the previous one (compare with 2022-02-08). (C) 2022-08-22 CT showed that after 17 cycles of treatment, the primary tumor in the right lobe of liver shrank (2.6x1.3cm in size), some intrahepatic metastases in the hepatic portal vein area, hepatogastric ligaments, and angina angina area shrank, and the abdominal paraaortic lymph nodes shrank compared with the previous time (compared with 2022-05-13).

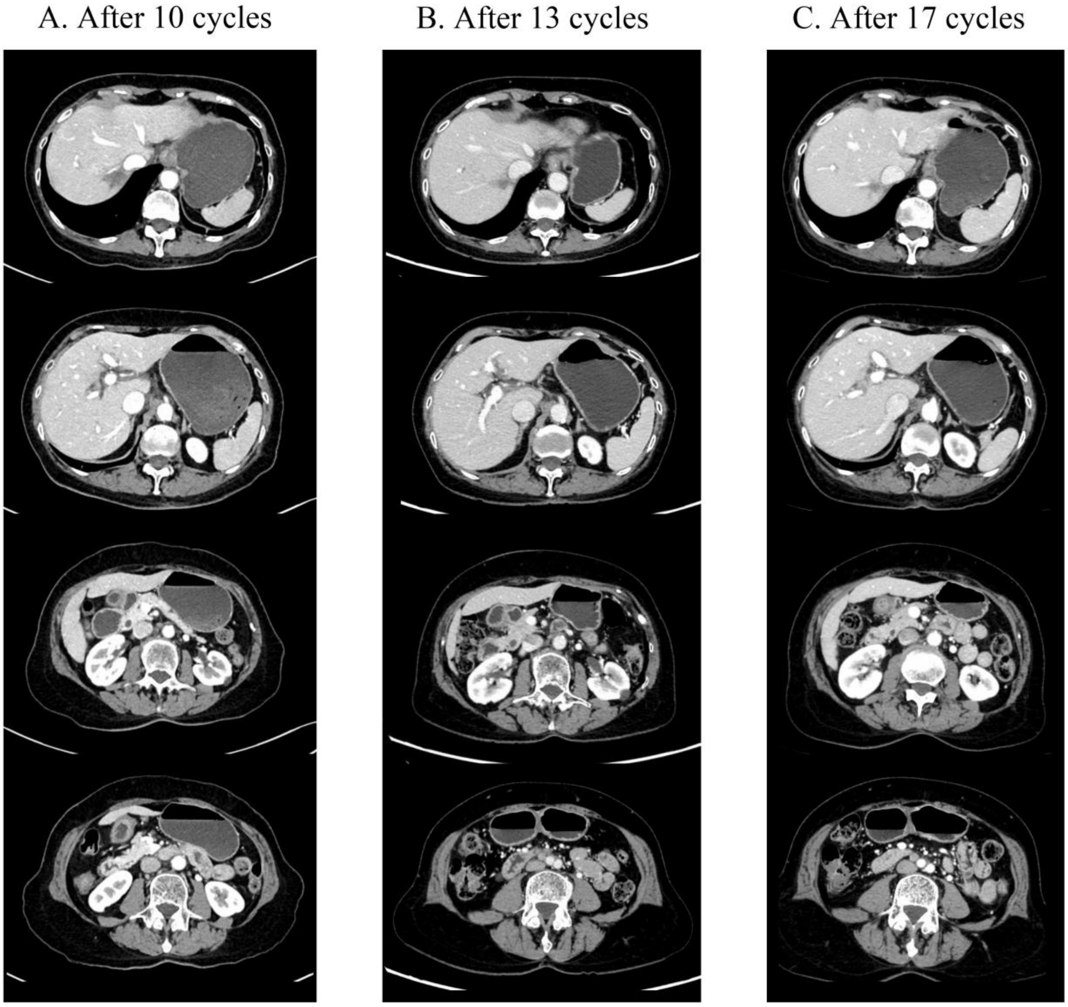

Supplement: Supplementary file 2 [file Image1.pdf]
